# Posterior Capsule Opacification and Nd-YAG rates evaluation in a large series of pseudophakic cases

**Published:** 2017

**Authors:** Ioana Madalina Iliescu, Maria Alexandra Constantin, Cristina Cozma, Ozana Manuela Moraru, Cristian Mircea Moraru

**Affiliations:** * Oculus Eye Clinic, Bucharest, Romania

**Keywords:** IOL design, IOL material, Nd-Yag rates, posterior capsule opacification

## Abstract

****Purpose**:**

To evaluate the influence of Intraocular Lens (IOL) material and design on Posterior Capsule Opacification (PCO) and Neodymium-YAG (Nd-YAG) rates in eyes implanted with different Posterior Chamber Intraocular Lenses (PC IOLs) designs at the end of uncomplicated cataract surgeries.

****Setting**:**

Oculus Eye Clinic, Bucharest, Romania.

****Design**:**

Retrospective, observational study.

****Methods**:**

This study comprised 4805 eyes operated for cataract in 2012 and 2013 with a post-operative average follow up of 40 ± 6,15 months (27-54 months). The PCO and Nd-YAG rates were recorded and compared among different IOL materials and designs and among different pathology groups.

****Results**:**

From 4805 IOLs implanted, 2560 (53,27%) were hydrophilic and 2245 (45,73%) hydrophobic, 2937 (61%) were aspherical and 1868 (39%) spherical. We found statistical significant differences in the PCO and Nd-YAG rates between hydrophilic (18% and 14% respectively) and hydrophobic lenses (4% and 2% respectively) (p<0.0001). There were also statistically significant differences in the sub-group of hydrophilic aspheric IOLs, finding lower PCO and Nd:YAG rates with the C-loop haptics configuration (12,6% and 3,3% respectively) compared with the broad optic/ haptic junction (29,75% and 24,73% respectively) (p<0.001). No statistically significant differences on PCO and Nd:YAG rates were found for the different associated pathologies (p>0.05).

****Conclusions**:**

Hydrophilic lenses showed statistically higher PCO and Nd:YAG rates than hydrophobic lenses. In contrast, the optic asphericity and the associated pathologies had no influence on the PCO and Nd:YAG rates. IOL design and material seem to be the main characteristics influencing PCO and Nd-YAG rates.

****Abbreviations**:**

LECs = lens epithelial cells

## Introduction

Cataract is a common and significant cause of visual impairment [**1**]. During surgery, it is impossible to mechanically remove all lens epithelial cells (LECs) from the capsular bag. The remaining equatorial LECs will migrate, undergo metaplasia, and form posterior capsule opacification (PCO).

PCO causes light scatter within the visual axis and produce visual disability [**2**]. PCO is considered the most common reason for reduced visual acuity after cataract surgery in otherwise healthy eyes [**3**]. PCO also degrades various aspects of visual function, including contrast sensitivity [**4**-**6**], glare disability [**7**-**9**], color vision and stereoscopic vision [**10**]. The treatment for PCO is Neodymium:YAG (Nd:YAG) laser capsulotomy which, although considered safe, may lead to some complications, including intraocular lens (IOL) damage, intraocular pressure (IOP) elevation, cystoid macular edema or retinal detachment [**11**].

Studies have shown a correlation between PCO rates and IOL characteristics, such as edge design [**12**] and optic material [**13**,**14**]. Also, creating a conti bnuous, centered and perfect sized anterior capsulorhexis can influence PCO occurrence [**15**]. 

In our study, we objectively quantified and compared PCO and Nd-YAG rates for all Posterior Chamber (PC) IOLs implanted in eyes operated for cataract in 2012 and 2013 in Oculus Eye Clinic, Bucharest. We recorded the PCO and Nd:YAG rates for hydrophilic/ hydrophobic and spherical/ aspherical IOL groups and evaluated the influence of IOLs characteristics on PCO and Nd:YAG rates. We also evaluated all the ocular and nonocular concurent pathology and their influence on PCO rates.

## Methods

Patients with uneventful cataract surgeries and no postoperative complications who were operated in 2012 and 2013 at the Oculus Eye Clinic, Bucharest, Romania, were included in this retrospective observational clinical trial. The study was approved by the Ethics Committee, Oculus Eye Clinic, Bucharest (02/ 2016) and registered with the Romanian National Agency of Drugs and Medical Devices. Our study is in agreement with “human and animal rights” and respects the Helsinki Declaration of 1975 revised in 2000 and 2008.

Patients’ charts were included in the study after examining all surgeries reports and postoperative information stored in the 2012-2013 Oculus Registry and only the charts that complied with the inclusion/ exclusion criteria were selected. 

The inclusion criterion was all uncomplicated eyes operated for cataract from January 2012 to December 2013. Exclusion criteria were eyes with ocular preoperative associated inflammatory pathology (uveitis) or trauma, complicated intraoperative cases with posterior capsular rupture, complicated postoperative cases: endophthalmitis, inflammation of different etiology of anterior or posterior pole or those with no follow-up visits. 

All surgeries were performed under topical anesthesia by five surgeons using ultrasound technology (INFINITI® Vision System, Stellaris PC or CENTURION® Vision System) followed by the polishing of the posterior capsule and the implantation of it in the bag PC IOL (of in the bag- without it). A small percentage (1.19%) of eyes had a capsular tension ring implanted addressing the zonular laxity. Few surgeries (1%) were followed by same session intravitreal injection of either Bevacizumab or Triamcinolone Acetonide for Diabetic Macular Edema (DME) or wet Age Related Macular Degeneration (AMD). Some surgeries (3,5%) were performed by using the femtolaser technology (LenSx® Laser System). Each patient received a PC IOL spherical or aspheric, hydrophilic or hydrophobic. 

All the patients received the same postoperative treatment: antibiotic and steroid fixed combination five times a day for two weeks, followed by steroid drops three times a day for another two weeks.

The follow-up examinations were at first day, first month, and 3 months to one year postoperative, depending on ocular status. Further examinations (at every 3, 6 or 12 months) up to over 4 years (max 54 months) of follow-up were scheduled dictated by ocular features, referring the doctors’ requests or the patients’ visual complaints/ demands.

The best distance and near corrected visual acuity (VA), anterior and posterior segment dilated slip lamp examination and tonometry were evaluated at each follow-up visit. The PCO was evaluated subjectively with the retro illumination of the slit lamp. PCO was considered when Elschnig’ Pearls or fibrosis could be noticed on the posterior capsule correspondent to the capsulorhexis opening.

Nd:YAG laser treatment was performed when BDCVA decreased at least 20% due to PCO occurrence. 

PCO and Nd:YAG rates were evaluated for the different IOL material and design groups included in the present study. 

Additionally, cases were assigned into different pathology groups and the PCO rates were recorded accordingly to the ocular or non-ocular pathologies.

**Statistical analysis**

Statistical analyses were computed with SPSS for Windows software (version 18.0, SPSS, Inc.) and Pearson Chi-Square test was applied when comparing rates for hydrophilic/ hydrophobic, spherical/ aspheric IOLs. Statistical significance was considered when p<0.05. 

## Results 

**Fig. 1:**
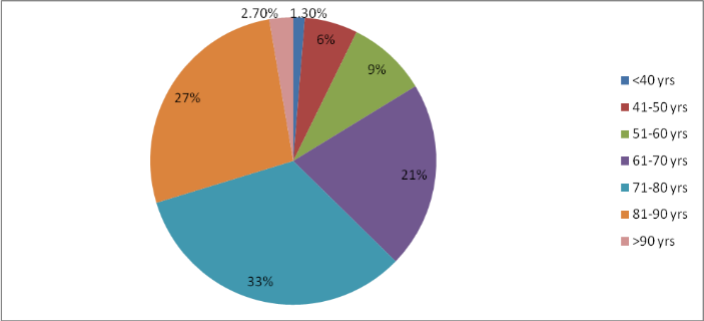
Patient age distribution

The current study comprised 4805 eyes from 3494 patients with a mean follow-up of 40 ± 6.15 months (range from 27 to 54 months). There were 1810 (38%) males and 2995 (62%) females. The mean age at the last follow-up was 73 ± 12 years (range 21 to 98 years) with 80% of the patients between 60 to 90 years old. The patient age distribution is plotted in **Fig. 1**.

All IOLs implanted during 2012 and 2013 at the Oculus Eye Clinic in Bucharest were included in the present study. **Table 1** shows the IOLs evaluated and their pertinent characteristics. A total of 4805 IOLs were implanted, of which: 2937 (61%) aspherical, 1868 (39%) spherical, 2560 (53.27%) hydrophilic and 2245 (45.73%) hydrophobic.

**Table 1:** Type of lenses implanted and their characteristics

LENS TYPE	%	No of cases	Manufacturer	Material	Design	Haptic angulation	Optics
Acri.Tec 47LC	1%	7	Acri. Tec	Hydrophilic with cover	1 piece with loops	Plate haptics	Aspheric
Acri.Tec 47S	4%	17	Acri. Tec	Hydrophilic with cover	1 piece with loops	Plate haptics	Spherical
Adapt-AO	4%	210	B&L	Hydrophilic	1 piece Square-edges	One-piece 4 closed loops 0° angulation	Aspheric aberration -free
Akreos- Adapt	1%	6	B&L	Hydrophilic	1 piece Square-edges	One-piece 4 closed loops 0° angulation	Spherical
Aspheric Ophtec	02%	1	Ophtec BV	Hydrophobic	1 piece with loops	2 closed loops 5°	Aspheric
Bioline Yellow	6%	273	iMedical	Hydrophilic	1-piece square edges	C-loops 0°	Spherical
B-Lens	2%	108	Hanita	Hydrophilic	1-piece square edges	C -loops 5°	Spherical
CT 47LC	2%	96	Zeiss	Hydrophilic	1-piece square edges	C-loops 0°	Aspheric
CT 47S	9%	430	Zeiss	Hydrophilic	1-piece square edges	C-loops 0°	Spherical
CT Xtreme D	04%	2	Zeiss	Hydrophilic	1-piece square edges	C-loops 0°	Spherical
Dr Schmidt	4%	19	HumanOptics	Hydrophilic	1-piece square edges	C Haptic 0°	Spherical
MA60AC	4%	17	Alcon	Hydrophobic	3-pieces sharp-edges	C-loops 10°	Spherical
MA60MA	1%	69	Alcon	Hydrophobic	3-pieces sharp-edges	C-loops 5°	Spherical
MC60T	02%	1		Hydrophobic			
MI60	17%	829	B&L	Hydrophilic	1-piece	4 haptics Slender 8°	Aspheric
MJ 14	4%	21	B&L	Hydrophilic	1-piece sharp edges	4 haptics Slender 8°	Aspheric
MX60	3%	124	B&L	Hydrophobic	1-piece post square edges	Modified C	Aspheric
MX60T	1%	54	B&L	Hydrophobic	1-piece post square edges	Modified C	Aspheric Toric
Ophtec Monomax PC550	6%	299	Ophtec BV	Hydrophilic	1-piece post square edges	C-loop 5°	Spherical
RAFI FOLDABLES	1%	51	RAFI Systems Inc	Hydrophilic	1-piece square edges	C-loop 0°	Spherical
SA60AT	7%	344	Alcon	Hydrophobic	1-piece square edges	C-loop 0°	Spherical
SeeLens	4%	191	Hanita	Hydrophilic	1-piece square edges	C-loop 5°	Spherical
SN60AT	1%	41	Alcon	Hydrophobic	1-piece square edges	C-loop 0°	Spherical
SN60WF	22%	1080	Alcon	Hydrophobic	1-piece square edges	C-loop 0°	Aspheric
SN6AD1	5%	256	Alcon	Hydrophobic	1-piece square edges	C-loop 0°	Aspheric multifocal
SN6ATx	5%	252	Alcon	Hydrophobic	1-piece square edges	C-loop 0°	Aspheric Toric
SND1TX	12%	6	Alcon	Hydrophobic	1-piece square edges	C-loop 0°	Aspheric multifocal Toric
XLSTABI SKI	02%	1	Zeiss	Hydrophilic	1-piece square edges	10°	Aspheric
Total	100%	4805					

The mean PCO and Nd-YAG rates for the hydrophilic group were 18% and 14%, respectively and for the hydrophobic group, they were 4% and 2%, respectively, finding a statistical significant difference between both groups (p<0.0001). We also found a statistical significant difference between the Nd-YAG rates of the spherical (3.8%) and aspheric lenses (10%) (p=0.000), but these spherical/ aspheric lenses groups showed large differences in the distribution of the lens types. We further divided these two large groups in sub-groups following material and lens optic/ haptic design.

The distribution of lenses in these sub-groups with their PCO and Nd-YAG Rates are shown in **Table 2**. No statistically significant differences were found between hydrophobic aspheric (C-Loop) and hydrophobic spherical IOLs (C-Loop) for PCO (p>0.05) and Nd:YAG rates (p>0.05). Regarding the hydrophilic IOL group, there were statistical significant differences between aspheric and spherical IOLs for PCO (p<0.0001) and Nd:YAG rates (p<0.0001). However, in the sub-group of hydrophilic aspheric IOLs, 89% of the lenses had a broad optic/ haptic junction and only 11% a C-Loop design in contrast to the C-loop haptic configuration found in all hydrophilic spherical lenses.

**Table 2:** The subgroup of lenses with their Posterior Capsule Opacification (PCO) and Nd-YAG rates

TYPE OF LENSES	No (% of total)	SUBTYPE	No (% of total)	PCO Rate	Nd-YAG Rate	Haptic Configuration	PCO Rate	Nd-Yag Rate
SPHERICAL LENSES	2937 (61%)	HYDROPHILIC	1164 (39.6%)	27.68%	22.3%	Broad optic/ haptic junction (89%)	29.75%	24.73%
						C-Loop (11%)	12.6%	3.3%
		HYDROPHOBIC	1773 (60.4%)	4.02%	2%	C-Loop (100%)	4.02%	2%
	1868 (39%)	HYDROPHILIC	1396 (74.73%)	7.64%	5.1%	C-Loop (100%)	7.64%	5.1%
		HYDROPHOBIC	472 (25.27%)	5.29%	1.9%	C-Loop (100%)	5.29%	1.9%

Analyzing the PCO and Nd:YAG rates for the sub-group of hydrophilic aspheric IOLs with different haptics configuration, we found statistically significant differences (p<0.001) for the PCO and Nd:YAG rates between the group of IOLs with broad optic/ haptic junction (29.75% and 24.73% rates, respectively) and C-Loop haptics (12.6% and 3.3%, respectively). In contrast, comparing the PCO and Nd:YAG rates of the hydrophilic aspheric sub-group with C-loop configuration (12.6% and 3.3%, respectively) versus the hydrophilic spherical sub-group with the same haptics configuration (7.64% and 5.1% rates, respectively), no statistically significant differences were found (p>0.05). 

The study also followed concurrent ocular and general pathology and their influence on PCO and Nd-YAG rates. **Table 3** shows the distribution of patients for each pathology group.

**Table 3:** Distribution of patients for each pathology group

OCULAR PATHOLOGY	GENERAL PATHOLOGY		
Age Related Macular Degeneration 21% (n=992)	Type II Diabetes 15% (n=734)	Oral medication 69%	No Diabetic Retinopathy 80%
			Background Diabetic Retinopathy 16%
Glaucoma 14% (n=656)		Insulin 17%	Proliferative Diabetic Retinopathy 3%
Pseudoexfoliative Syndrome 8% (n=370)		Diet 14%	Diabetic Maculopathy 1%
Intravitreal Injections 1% (n=40)	General Cortisone Treatment ~1% (n=59)		

**Table 4** shows the PCO rates for each pathology group with the additional information of the percentage of hydrophilic lenses per group. There were no statistically significant correlations between the ocular associated pathologies and PCO rates (p>0.05).

**Table 4:** PCO rate for each pathology group

PATHOLOGY	Rate of PCO
Diabetes (n=734)	**6%** (n=46) - 47% hydrophilic lenses
Age Related Macular Degeneration (n=992)	**12%** (n=123) - 62% hydrophilic lenses
Glaucoma (n=656)	**16%** (n=108) - 55% hydrophilic lenses
Pseudoexfoliation Syndrome (n=370)	**12%** (n=45) - 68% hydrophilic lenses
General Cortisone treatment (n=59)	**2%** (n=1) - 23% hydrophilic lenses

## Conclusions

In the current study, a total of 4805 implanted IOLs were evaluated. The incidence of the PCO and Nd:YAG rates were assessed after a mean of 40±6.15 months of follow up (from 27 to 54 months). The analysis showed that PCO and Nd:YAG rates were statistically significantly higher in those eyes implanted with hydrophilic IOLs than with hydrophobic IOLs. Therefore, IOL material seems to be a determinant factor for PCO occurrence and need of Nd-YAG laser treatment after uneventful cataract surgery. Our results were in accordance with several studies that have shown a correlation between PCO rates and optic material [**13**,**14**]. Hydrophilic IOLs have been associated with higher rates of PCO than hydrophobic IOLs [**16**,**17**]. 

Regarding the IOL characteristics/ material, many studies comparing square-edged hydrophobic and hydrophilic acrylic IOLs [**17**-**20**] have shown that hydrophilic IOLs perform less favorably than hydrophobic IOLs in terms of PCO prevention. 

Regarding the IOL asphericity, we did not find any significant correlation for PCO and Nd:YAG occurrence. Besides, that hydrophilic aspheric IOLs sub-group showed statistically significant higher PCO and Nd:YAG rates than hydrophilic spherical IOLs, these results being mainly attributed to the fact that in the sub-group of hydrophilic aspheric IOLs, 89% of the lenses had a broad optic/ haptic junction with high PCO and Nd-YAG rates and only 11% showed a C-Loop design in contrast to the C-loop haptic configuration found in all hydrophilic spherical lenses. Our statistical analysis confirmed that, for the hydrophilic lenses, we found significantly higher PCO and Nd:YAG rates for the IOLs with the broad optic-haptic junction than for those with the C-loop configuration without any correlation to the asphericity. In contrast, when we compared the PCO and Nd:YAG rates of the hydrophilic aspheric sub-group with C-loop configuration (12.6% and 3.3%, respectively) versus the hydrophilic spherical sub-group with the same haptics configuration (7.64% and 5.1% rates, respectively), no statistically significant differences were found (p>0.05).

The broad gap in the squared-edged barrier at the broad optic–haptic junction of an IOL has been described as the Achilles heel in PCO prevention as it provides a point of migration for LECs from the equatorial region of the capsule onto the central posterior capsule [**21**].

We noticed a lot of variability in PCO and Nd:YAG rates among the different hydrophilic IOL models evaluated. These differences might be due to the differences in the haptics and edges design. In contrast, regarding to the hydrophobic IOLs group, all the lenses showed very similar PCO and Nd:YAG rates, with very similar IOL design characteristics.

Concerning the different pathology groups, our study found no statistically significant correlations between the ocular associated pathologies and PCO rates (p>0.05).

Being a retrospective analysis conducted on a large number of eyes, this study had some limitations. One limitation was that the groups of IOLs formed by model were not equal in size and the distribution of IOLs among different pathology groups was not the same. Another limitation was the use of the CTR or Femtolaser in some particular cases or some intravitreal injections done at the end of the surgeries.

Despite the minimal limitations, our study had many strong points. We managed to include a large sample of eyes with a high diversity of IOLs implanted and we followed these eyes for a long period of time. Being also able to perform Nd-YAG laser treatment on site, all our patients who developed PCO and needed to be treated, could come to the clinic and receive proper care.

Our study also showed that not only the material of the IOL was a determinant factor for PCO formation, but also the design of the optic/ haptic. The C-Loop design performed statistically better in terms of PCO prevention compared to the broad optic/ haptic junction configuration despite same hydrophilic material and similar edges configuration. Our analysis led to the additional conclusion that asphericity or co-existing ocular or non-ocular pathology did not influence the PCO occurrence.

**Acknowledgments:**

Dr. Iliescu, Constantin and Cozma were supported by a research Grant from Alcon Laboratories, Inc. (Alcon Romania S.R.L., located at 301-311 Barbu Văcărescu Blvd., Lake View Building, 5th floor, district 2, Bucharest, Romania, IIT Grant No. 20949341).

No authors have any financial interest in a product, method, or material presented in the article.

The article was presented in the following meetings:

IIIrd Congress of the Romanian Society of Cataract and Refractive Surgery - Eforie Nord, Romania, 23-26 June 2016; 

The XXXIV Congress of the European Society of Cataract and Refractive Surgeons- Copenhagen, Denmark, 10-14 Sept 2016;

XVth National Congress of the Romanian Society of Ophthalmology – Sinaia, Romania, 5-8 October 2016.
